# Association between neurological and rheumatological manifestations in vitamin D deficiency and vitamin D levels

**DOI:** 10.12669/pjms.293.3447

**Published:** 2013

**Authors:** Faiza A. Qari, Tariq A. Nasser

**Affiliations:** 1 Faiza A. Qari, FRCP, ABIM, Department of Internal Medicine/Endocrinology, King Abdulaziz Medical City, Jeddah, Saudi Arabia.; 2 Tariq A. Nasser, ABIM, Department of Internal Medicine/Endocrinology, King Abdulaziz Medical City, Jeddah, Saudi Arabia.

**Keywords:** Vitamin D deficiency, Vitamin D, Proximal myopathy, Bone profile

## Abstract

***Objective:*** We aimed to investigate the associations between the neurological manifestations of vitamin D deficiency and bone profile as well as the levels of 25-hydroxyvitamin D.

***Methodology:*** We conducted a case series on patients with vitamin D deficiency who were followed up at King Abdulaziz Medical City, Jeddah between January 2010 and December 2011. We collected patients’ demographic data and gathered information on etiological factors for vitamin D deficiency as well as clinical presentations (typical, neurological and rheumatological) and radiological findings. The t-test was used to determine whether there was an association between the neurological manifestations of vitamin D deficiency and vitamin D levels and bone profile.

***Results:*** We enrolled 60 patients with vitamin D deficiency. Of these, 44 (73.3%) had neurological presentations, namely progressive muscle weakness and proximal weakness, which was observed more often than distal weakness. In addition, gait disturbances were observed in 61.7% of all patients with neurological and rheumatological presentations. There was no significant association between neurological and rheumatological manifestations and bone profile or vitamin D levels. We found a significant association between difficulty in walking and the levels of serum calcium and phosphate (*P* = 0.043 and 0.037, respectively).

***Conclusion:*** Neurological and rheumatologic manifestations of vitamin D deficiency are not associated with 25-hydroxyvitamin D levels or bone profile.

## INTRODUCTION

One of the most important causes of osteomalacia is vitamin D deficiency.^1^ Vitamin D3 (cholecalciferol) is synthesized non-enzymatically in the skin from 7-dehydrocholesterol during exposure to ultraviolet rays in sunlight.^2^ The etiological causes of vitamin D deficiency are due to limited exposure to sunlight combined with lack of vitamin D-fortified foods or malabsorption.^3^ Alternatively, impaired hydroxylation of vitamin D in the liver or kidney can prevent the metabolism of the vitamin into its physiologically active form.^4^ Although sunshine is abundant in Saudi Arabia throughout the year, the local population avoids exposure to sunlight in order to stay cool and for socio-cultural reasons.^5^^,^^6^ A vast majority of the population uses headgear, and Saudi women in particular have adopted a strict clothing code; they cover most of their bodies when they leave their homes. This, in addition to the effects of high parity and prolonged breastfeeding, puts them at high risk of developing vitamin D deficiency.

The typical clinical manifestations of vitamin D deficiency include nonspecific backache, bone pain, and generalized body ache.^7^ Progressive proximal muscle weakness, gait disturbances, carpopedal spasm, and bone deformity are atypical manifestations of this deficiency.^8^ As patients with osteomalacia might have severe neurological presentations even in cases of mild vitamin D deficiency and bone profile, it is important to conduct an observational study in clinical practice in order to assess the association between vitamin D levels and disease presentation, especially for atypical neurological and rheumatological presentations.^9^ Thus, the objective of this study was to investigate the correlation between the neurological and rheumatological manifestations of vitamin D deficiency and the levels of 25-hydroxyvitamin [25(OH) D] and bone profile (calcium, phosphate, magnesium and alkaline phosphatase).

## METHODOLOGY

We conducted a case series study on patients who were followed up at the Endocrinology Outpatient Clinics of King Abdulaziz University Hospital, Jeddah from January 2010 to December 2011. Consent was obtained from the participants prior to their inclusion in the study. The study was approved by the Biomedical Ethics Research Committee of King Abdulaziz University. 

We included all patients with a diagnosis of vitamin D deficiency, which was based on findings of low serum 25(OH) D levels and not on results of bone profile.

A consultant endocrinologist evaluated all the patients enrolled in the study. For all patients, we recorded the following information: detailed symptoms and signs, dietary history, duration and severity of illness, associated illnesses, sun exposure of <60 minutes per week, renal disease, gastrointestinal tract disease, and use of anticonvulsants such as diphenylhydantoin sodium. Biochemical data, radiological findings, and the treatments administered (vitamin D and calcium supplements) were also recorded.

For the purpose of this study, neurological manifestations were defined as reports of paresthesia, tetany, and progressive proximal myopathy including inability to walk and get up from a squatting position and waddling gait. Rhematological manifestations were defined as myalgia, arthralgia, and fractures.

***Statistical Analysis:*** Analysis was carried out using the Statistical Package for Social Sciences (SPSS), version 16. The t-test was used to analyze the association between the neurological and rheumatological manifestations of vitamin D deficiency and 25(OH) D levels and bone profile.

## RESULTS

In total, 60 patients were enrolled in the study. The mean ± SD age of the patients was 31 ± 13.7 years (range, 13–60 years). There were 15 men and 45 women, with a men to women ratio of 1:4.

The most frequent typical manifestation of 25 (OH) D deficiency was bone pain, which was observed in 48 patients (80%). Other typical presentations included backache (56%), mylagia and bone tenderness (72%). 

There were different neurological clinical manifestations of 25 (OH) D deficiency. Progressive muscle weakness was observed in 73.0% of the patients, who had an atypical presentation; proximal weakness was more common than distal weakness. Amongst the patients with proximal weakness, six (20%) had severe symptoms, which led to wheelchair-bound states. The weakness was moderate in 22 patients (42%). Patients with severe symptoms had significant restriction of activities of daily living with inability to get up from a squatting position and waddling gait. Gait disturbance was noted in 61.7% of patients who had atypical presentations. These patients were treated with vitamin D supplements and calcium, and 46% of them showed a dramatic response to treatment. The least frequent neurological clinical manifestations observed in our study group included tetany and paresthesia (9% and 18%, respectively.

The commonest etiology of 25 (OH) D deficiency was limited sun exposure in most of the cases. Vitamin D deficiency of dietary and celiac origin were observed in 5% and 14% of the cases, respectively. Miscellaneous causes included anticonvulsant medication (phenytoin), oncogenic osteomalacia (tumor induced osteomalacia), vitamin D-resistant rickets, and chronic kidney diseases in 5 cases. These patients had renal osteodystrophy and osteomalacia in the form of low or normal calcium levels, high phosphate levels, metabolic acidosis, and low 1,25-dihydroxyvitamin D3 levels.

The radiological findings of patients with 25(OH) D deficiencies were osteopenia (60%), followed by Looser’s zone (43%). Other radiological findings were bowed legs and true fractures. In 10% of the cases, radiological findings were normal.

The bone profile of patients with 25(OH) D deficiencies is shown in Table-I, while Table-II shows the correlation between the levels of 25(OH) D and bone profile (calcium, magnesium, phosphate, alkaline phosthatase and PTH) with the presence or absence of neurological presentations in the subjects.

There was no significant association between low levels of 25(OH) D and neurological manifestations of vitamin D deficiency or between deficiencies in 25(OH) D and bone profile. The only significant association observed was between difficulty in walking and the levels of calcium and phosphate (*P* = 0.043 and 0.037, respectively) (Table-II).

## DISCUSSION

Vitamin D deficiency is the most common cause of osteomalacia, and it generally results from limited exposure to sunlight, dietary deficiency, or celiac disease.^3^^,^^4^ Limited sun exposure occurs in homebound people, dark-skinned individuals, heavy sunscreen users, and those who have limited exposure for social, cultural, or health reasons. In the case of our patients, who all resided in Saudi Arabia, excessive clothing and covering of the skin was the most common cause of limited exposure to sunlight (68%). The etiology of vitamin D deficiency in our patients was related to a combination of the lack of sun exposure due to wearing of the traditional dress, dark skin color, and poor dietary habits.^6^ Celiac disease^10^ was the second common cause of osteomalacia in our study group (20%). In addition, other causes included anticonvulsant therapy (phenytoin),^11^^,^^12^ tumor-induced osteomalacia,^13^^,^^14^ familial vitamin D-resistant rickets, and renal osteodystrophy^15^_. _However, our observation that decreased intake of food^16^ contributed to 25(OH) D deficiency in only 5% of our study population, which could be the result of our failure to record specific details upon history taking. 

**Table-I:** Laboratory data of the study subjects

Laboratory test levels*	*Mean * *±* * SD*	*Minimum*	*Maximum*
Calcium	2.001 ± 0.21	1.19	2.39
Phosphate	2.14 ± 7.7	0.35	4.4
Magnesium	0.77 ± 0.142	0.45	1.30
Creatinine	76.85 ± 52	40	432
HCO_3_	22 ± 2.55	16	28
Vitamin D3	18.6 ± 22.54	**10**	79

Abbreviations: HCO_3_, bicarbonate; PTH, parathyroid hormone

*Reference range for laboratory tests: calcium (2.12–2.52 mmol/L); creatinine (53–115 µmol/L); HCO_3_ (21–28 mmol/L); magnesium (0.7–1.0 mmol/L); phosphate (0.81–1.5 mmol/L); vitamin D3 (75–200 nmol/L). PTH (11 - 54 pg/mL).

**Table-II:** **Association** between 25(OH) D levels and bone profile with the presence or absence of atypical neurological and rheumatlogical presentations in study subjects*

*Character*	*Paresthesia*			*Tetany*			*Difficulty in walking*			*Deformity*		
	*+*	*–*	*T test*	*+*	*–*	*T test*	*+*	*–*	*T test*	*+*	*–*	*T test*
Calcium level mmol/L	2.1	2.0	0.9	1.89	2.01	0.54	2.00	2.0	0.043	2.0	2.00	0.6
Phosphate mmol/L	0.79	0.7	0.29	0.69	0.7	0.97	0.65	0.7	0.037	0.7	0.7	0.5
Magnesium mmol/L	0.8	0.8	0.42	0.81	0.8	0.32	0.73	0.8	0.2	0.9	0.8	0.3
Alkaline phosphatase ul/L	350	472	0.91	334	375	0.36	350	351	0.8	386	351	0.6
Vitamin D3 level nmol/L	10	10	0.89	17	10	0.21	10	10	0.32	10	10	0.3
PTH level pg/ml	268	247	0.92	287	248	0.52	287	286	0.62	304	286	0.1

*The signs (+) and (–) denote the presence and absence of neurological clinical manifestations in correlation with laboratory level values of bone parameters (calcium, phosphate, magnesium, alkaline phostase) and level of vitamin D3 and PTH l.

T-test showed if there is a significant correlation between the neurological manifestations with a level of laboratory values of bone profile and vitamin D3 level

Reference range for laboratory tests: alkaline phosphatase (50–130 ul/L); calcium (2.12–2.52 mmol/L); creatinine (53–115 µmol/L); HCO_3_ (21–28 mmol/L); magnesium (0.7–1.0 mmol/L); phosphate (0.81–1.5 mmol/L); PTH (1.6 –6.9 pg/mL); vitamin D3 (50–125 nmol/L). PTH (11 - 54 pg/mL)

***Clinical presentations:*** Previously reported non-specific symptoms, such as back pain,^17^ arthralgia,^18^ and bone tenderness^19^ were observed in almost all patients. It was apparent that our patients with vitamin D deficiency may not present with typical clinical presentations of osteomalacia; however, they may present with an atypical neurological presentation such as severe proximal myopathy.^20^^,^^21^ This may be accompanied with morbidities, including waddling gait and difficulty in walking, which were reported in 73% and 61% of the cases, respectively.^22^

Hypophosphatemia, high levels of parathyroid hormone, and low levels of calcitriol are the mechanism of myopathy.^23^ Experimental studies have also shown that skeletal muscle contains vitamin D receptors that specifically bind to 1, 25(OH) D3 and modulate various transcription factors in muscle cells.^24^ The factors that contributed to muscle weakness in our patients were due to neurotoxic effects, which resulted from muscle cell proliferation and differentiation into mature muscle fibers as elevated parathyroid hormone levels.^25^

Based on radiological findings, reduced bone density with thinning of the cortex (osteoporosis) was the most common finding (60%) in our study, which is in line with the results of a recent study.^26^ Looser’s zones (pseudo-fractures) are the characteristic radiologic findings in osteomalacia;^27^ these appear as fissures or narrow radiolucent lines with sclerotic borders lying perpendicular to the cortical margins. These were observed in 45% of our patients.^28^ However, we found no significant statistical correlation between radiological features and bone parameters and vitamin D levels.

***Correlation between 25(OH) D deficiency and neurological clinical presentations:*** In this study, we investigated the correlation between the bone profile, 25(OH) D levels, parathyroid hormone levels and atypical neurological symptoms (Table-II).^29^ As a result, we concluded that patients with low 25(OH) D levels may have neurological presentations such as difficulty in walking and proximal myopathy;^30^ however, there was no significant association between neurological and rheuamatological manifestations and bone profile and 25(OH) D levels.^29^^,^^30^ The absence of a significant association between severe symptoms and low 25(OH) D levels can be explained by the fact that even at low 25(OH) D levels, there is sufficient 1, 25 (OH) 2 D3 to maintain homeostasis. Indeed, there may be (over) active conversion of 25 (OH) D to 1,25 (OH)_2_ D3 to maintain bone mass. However, further studies are required to elucidate the exact nature of this mechanism.

***Response to Treatment*** *:* Patients initially showed significant clinical improvement after treatment with vitamin D and calcium.^31^ However, follow up was inefficient because we could not obtain relevant information for assessing the clinical and biochemical response to long-term treatment. This made it impossible to accurately assess their response to treatment. However, it is worth noting that the response to treatment in 46% of the patients with severe proximal myopathy was dramatic.^32^

Some of our wheelchair-bound patients also responded dramatically to treatment. After many months of disability, they were able to ambulate independently and function normally with simple medical treatment.^33^ However, the reason for treatment failure in the rest of the patients with myopathy was unclear. They were followed up by a neurologist and rheumatologist. Some underwent further investigations, such as electromyography and muscle biopsy for myopathy.^34^

***Limitations:*** This study has several limitations. First, we did not record specific dietary history, including the consumption of milk or milk products for all patients included in the study. Second, there was a lack of formal documentation of the response to treatment and follow up of biochemical changes.

## CONCLUSIONS

Although patients with vitamin D deficiency had neurological and rheumatological signs and symptoms, including difficulty in walking severe proximal myopathy, and fractures, we did not find any association between these and the levels of 25(OH) D or bone profile (calcium, magnesium, phosphate, and alkaline phosphatase). Further investigations are needed to explain why severe neurological presentations of vitamin D deficiency are not correlated with low levels of vitamin D. These observations are very important in clinical practice for the management of vitamin D deficiency.

## Notes

***Financial/nonfinancial disclosures:*** The authors have reported no significant conflicts of interest exist with any companies/organizations whose products or services may be discussed in this article.

***Funding support:*** None**.**
